# Knowledge, attitudes and practices on substandard and falsified medicines for human and animal use in Wakiso district, Uganda

**DOI:** 10.1080/20523211.2025.2564822

**Published:** 2025-10-06

**Authors:** David Musoke, Grace Biyinzika Lubega, Carol Esther Nabbanja, Filimin Niyongabo, Michael Obeng Brown, Elma Rejoice Banyen, Jody Winter, Claire Brandish, Kate Russell-Hobbs, Natasha Hamilton, Herbert Bush Aguma, Linda Gibson

**Affiliations:** a Department of Disease Control and Environmental Health, Makerere University School of Public Health, Kampala, Uganda; b Institute of Health and Allied Professions, School of Social Sciences, Nottingham Trent University, Nottingham, United Kingdom; c Department of Biosciences, School of Science and Technology, Nottingham Trent University, Nottingham, United Kingdom; d Pharmacy Department, Buckinghamshire Healthcare NHS Trust, Aylesbury, United Kingdom; e Department of Pharmacy, School of Health Sciences, Makerere University College of Health Sciences, Kampala, Uganda

**Keywords:** Substandard, falsified, medicines, knowledge, attitudes, practices, humans, animals, antimicrobial resistance, one health

## Abstract

**Background:**

Substandard and falsified medicines (SFMs) continue to pose a significant threat to public health globally. However, there is limited evidence on use of SFMs for both humans and animals particularly in low- and middle-income countries such as Uganda. The study assessed knowledge, attitudes and practices on SFMs for human and animal use in Wakiso District, Uganda.

**Methods:**

A cross-sectional survey that employed a structured questionnaire among 432 community members was conducted in Wakiso District. The questionnaire assessed knowledge, attitudes and practices on SFMs for human and animal use. Data was collected using the KoboCollect mobile application hosted on tablet computers. Univariate data analysis was conducted in Stata Version 14.

**Results:**

The majority of respondents (83%) stated that they had heard about SFMs although only 31% could correctly define them. Only 7% of the respondents accurately identified a falsified medicine despite 24% stating that they believed they could recognise SFMs. Almost two-thirds (62% and 60%) of the respondents disagreed that most human and animal SFMs respectively were as good as genuine medicines. Most of the respondents strongly agreed or agreed that SFMs could be very dangerous for humans (96%) and for animals (95%). Respondents reported having bought products they suspected were SFMs for use in humans (14%) and animals (24%). Seeking health worker advice on the medicine brand (40%) / getting medicine from a trustworthy pharmacy (34%) for humans; and seeking a veterinary officer’s advice for choosing the brand (43%) / getting medicine from a trustworthy veterinary pharmacist (29%) for animals were the most common measures respondents reported taking to ensure the medicine bought was genuine. Only 25% of the respondents mentioned informing a health worker and only 4% had reported suspicions of SFMs to the National Drug Authority.

**Conclusion:**

Despite commendable attitudes, there was generally limited knowledge and related poor practices regarding SFMs for both humans and animals. There is a need for key stakeholder engagement involving health and regulatory authorities in both human and animal medicine to increase awareness on SFMs to minimise the potential risks to health among the community.

## Background

Substandard medicines are medical products with either low quality standards and / or specifications that do not meet requirements as stipulated by regulatory bodies (WHO, 2018). Falsified medicines may include medical products with deliberately incorrect ingredients or quantities, or may have no active ingredient of international standards (Salami et al., 2023; WHO, 2018). Substandard and Falsified Medicines (SFMs) impact global population health in numerous ways (Nayyar et al., 2019; WHO, 2018). They are a major global health challenge because the low-quality standard and specification and / or falsification of the composition of the medical products renders them unsafe (Salami et al., 2023). Exposure to harmful, unnecessary chemicals can result in unwanted side-effects, and lack of effective treatment can result in worsening health conditions and even death. Current evidence suggests that SFMs impact on the global population health through increasing the risk of adverse reactions or side effects to consumers (WHO, 2018) who can remain ill for longer periods and may increase morbidity or mortality rates if left unchecked (Nayyar et al., 2019; Salami et al., 2023). Yet despite the risks that SFMs pose, the Organisation for Economic Cooperation and Development (OECD) reported that the global sales of these drugs were greater than $200 billion in 2016 (OECD, 2020) making it a lucrative business (Siva, 2010).

The risks associated with SFMs extend to other alarming global health issues such as antimicrobial resistance (AMR). There is evidence that AMR is exacerbated by the proliferation of SFMs as inadequate dosing can fuel the emergence of drug-resistant pathogens (Cavany et al., 2023; Dione et al., 2021). The use of SFMs contributes to the global rise of AMR which has not equated to the development of novel medicines that are available to treat resistant bacteria (Cavany et al., 2023). The use of SFMs poses significant risks to both human and animal health, leading to treatment failures, worsening diseases, and potentially death (WHO, 2017). In humans, they can cause adverse reactions, and strain healthcare systems (O'Neill, 2016). For animals, SFMs may result in ineffective treatments, prolonged suffering, and economic losses, affecting industries reliant on animal products and food security (Grace, 2015; Vidhamaly et al., 2022). The unchecked use of SFMs also raises ethical and legal concerns, often linked to criminal activities that undermine trust in healthcare systems (Attaran et al., 2012). In addition, these medicines can have environmental impacts, contaminating ecosystems, and posing broader threats to global health security (Kümmerer, 2009). Addressing this issue therefore requires coordinated global efforts, including stricter regulation, enforcement, public awareness, and international cooperation (WHO, 2017).

In Africa, and other low- and middle-income countries (LMICs), one in every ten medical products can be classified as SFMs (WHO, 2024). These findings are supported by a study that found the prevalence of SFMs to be 13% within the African region including Uganda (Ozawa et al., 2018). In addition, 42% of falsified medical products reported by the World Health Organization (WHO) between 2013 and 2017 were predominantly in African countries (WHO, 2018). This high usage of SFMs is mainly driven by either consumer or insufficient availability and access factors (Dione et al., 2021). The consumer driven factors encompass low purchasing power of clients and being unaware of the potential risks (Buckley & Gostin, 2013; Orubu et al., 2020). However, the supply and demand factors are driven by corruption in the pharmaceutical industries, weak technical capacity and quality assurance of medical suppliers, and poor supply chain managements (WHO, 2010, 2017). In addition, other factors such as lack of access to good quality healthcare services, poor governance, and weak law enforcement increase the risk of SFMs among the community (Orubu et al., 2020).

There have been efforts to find solutions to this complex but critical global health issue of SFMs (WHO, 2017). These efforts include regulatory standards and measures; governmental guidelines; research and development in pharmaceutical industries; proper supply chain management; awareness campaigns; and collaborations with WHO to have effective confiscation mechanisms for SFMs (Aminu et al., 2017). However, these efforts have not been successful largely because of having a fragmented approach and many requiring high-cost technologies (Aminu et al., 2017; Ozawa et al., 2018). To ensure there is a global workplan, WHO have set up a global mechanism for reporting SFMs within the supply chain. In addition, many countries, including those in sub-Saharan Africa such as Uganda are now being supported to formulate local policy and regulations of their medicinal products (Attaran et al., 2012; Hamilton et al., 2016), and provide training in their health sectors (WHO, 2019b). However, the current efforts focus on the supply-side of the medicinal products and health facilities without much consumer engagement. Consumers need to be educated and empowered to demand quality and genuine medicines. As such, research on SFMs that focuses on consumers’ experiences in human and animal health is limited (Isuga et al., 2022; Noun et al., 2021). It is paramount to understand how a multisectoral approach that involves various stakeholders including consumers (from both the human and animal sectors) might be useful for LMIC economies to tackle SFMs. In Uganda, evidence is sparse on SFMs from a consumer perspective. Our study therefore aimed to address this research gap by assessing the knowledge, attitudes and practices on SFMs for human and animal use in Wakiso district, Uganda.

## Methods

### Study design and setting

We conducted a cross-sectional study that employed a structured questionnaire among community members to assess knowledge, attitudes and practices on SFMs for human and animal use in Wakiso district, Uganda. Wakiso district is located in the central region of the country and partially encircles the capital city, Kampala. It shares borders with Nakaseke and Luweero districts to the North, Mukono district to the East, Kalangala district to the South, Mpigi district to the Southwest, and Mityana district to the Northwest. Wakiso was chosen for the study because it is the most populated district in Uganda with vast rural, peri urban and urban areas. The district has a population of 3,411,177 (UBOS, 2024) and is divided into two counties, Kyadondo and Busiro. The main economic activities in the district include business, agriculture, farming, and fishing.

### Sample size and sampling

A minimum sample size of 428 was calculated using a 95% Confidence Interval, 5% precision, non-response rate of 10% (WHO, 2021), and a prevalence of 50% given that knowledge on counterfeit medicines ranges from 30 to 93% (El-Dahiyat et al., 2021; Mhando et al., 2016; Sholy & Saliba, 2018). Data was subsequently collected from 432 respondents. The study employed multi-stage sampling at county, constituency, sub-county, parish, and village levels. Using random sampling, Busiro county was chosen from the 2 counties in the district. From the 5 constituencies in Busiro, purposive sampling was used to select Busiro North as it has a good representation of urban, peri urban and rural communities. Busiro North has 3 sub counties (Kakiri, Masulita and Namayumba) and 3 town councils (Kakiri, Masulita and Namayumba). Using random sampling, one parish was selected from each of the 3 sub-counties and town councils, and from each selected parish, 1 village was randomly selected. Mmanze, Bbembe, Lukoma, Kanzize, Kikubambanga and Kamuli were the final villages involved in the study. The required number of households per village was determined by dividing the sample size by the total number of selected villages. Therefore, 72 households were involved from each of the 6 villages. The households that participated in the study were selected systematically. The interval for selection of the households, which ranged from 2 to 10, was determined by dividing the approximate number of households in the selected village (as per the list of households obtained from the local council chairperson) by the required number of respondents per village. The initial households for the villages were randomly selected, and an interval was taken into consideration to select the next household. Only one respondent who was a health care decision maker, aged 18 years and above, and had lived in the household for more than 6 months was selected per sampled household. In cases where there was more than one eligible person, the respondent was selected randomly.

### Data collection

Six research assistants (RAs) were trained and oriented by the investigators to ensure that they were well versed with the study aim, methodology, and tool before data collection. The RAs were also equipped with various data collection skills such as probing and the appropriate way of recording responses. A research supervisor (FN), who was a graduate of Environmental Health Sciences with vast experience in research, ensured that all information was accurately collected and recorded during the data collection process. The questionnaire employed in the study contained 4 sections: 1 on socio-demographic characteristics such as age, gender, marital status, household income, presence of chronic illness, and ownership of animals; 2 on knowledge regarding SFMS using 9 questions which were dichotomised into three categories (yes, no and unsure); 3 on attitudes towards SFMs (9 questions for humans and 9 questions for animals) hence 18 questions where respondents had to state their level of agreement from strongly disagree, disagree, neutral, agree, and strongly agree; and 4 on practices regarding SFMs (humans and animals) such as purchasing frequency of medicines, source of medicine, ways of differentiating SFMs, measures taken to ensure authenticity of medicine, and reporting of SFMs to authorities. To identify SFMs, a laminated card with photos of Augmentin® packaging A and B was printed in colour and presented to respondents for identification (Supplemental Material). The questionnaire was developed based on previous research (El-Dahiyat et al., 2021; Isuga et al., 2022; Noun et al., 2021), and pre-tested in a village in Wakiso district that was not involved in the study. Data was collected in the local language (*Luganda)* which is most commonly used in Wakiso district following translation of the questionnaire from English. The data was entered through the KoboCollect application hosted on tablet computers.

B is an image of confirmed falsified Augmentin found in Uganda and Kenya in 2019. The product alert was first published by WHO in 2018. The falsified product can be identified by the atypical black logo of GlaxoSmithKline with white text instead of the orange logo with white text found on genuine product sold in Uganda (WHO, 2019a).

### Data management and analysis

Data was downloaded from the web-based KoboCollect software, a digital tool used for mobile data collection. The raw data was initially exported to Microsoft Excel for preliminary cleaning, which included checking for completeness, consistency, and accuracy of responses. Each categorical variable was reviewed to ensure consistent coding and labelling/ re-labelling. Following data cleaning, the dataset was exported to Stata Version 14.0 (StataCorp, Texas, USA) for statistical analysis. Given the descriptive nature of the study, the focus was on summarising individual variables independently to understand the distribution of responses within each category. Descriptive statistics were computed for all categorical variables. These included frequencies (n) and frequencies (percentages, %). The results were presented in tabular format to facilitate interpretation of the distribution patterns within the study population. The primary aim of this analysis was to describe the characteristics of the study sample and provide a foundational understanding of key variables relevant to the research objectives. No inferential statistical methods were applied at this stage, as the focus remained strictly on univariate descriptive analysis. All data were handled with strict adherence to confidentiality and data protection protocols. The cleaned and anonymised dataset was stored in encrypted files, accessible only to the core research team.

## Results

### Socio-demographic characteristics of respondents

The majority of respondents were female (64%), from rural settings (51%), and within the range of 31 to 50 years (47%). Two-thirds of the respondents were married (66%), and most had attained primary education (46%). Most respondents were farmers (40%), and on average earned between 27 to 135 USD per month (52%). Nearly half of the respondents owned animals (42%) among whom 89% reported that their animals suffered from seasonal illnesses (Table 1).

**Table 1.** Socio-demographic characteristics of respondents.

Variable	Responses	Frequency *n* = 432 (%)
Location description	Rural	221 (51)
	Semi-urban	139 (32)
	Urban	72 (17)
Gender	Male	155 (36)
	Female	277 (64)
Age (years)	18–30	117 (27)
	31–50	203 (47)
	>50	112 (26)
Marital status	Single	60 (14)
	Married	286 (66)
	Cohabiting	86 (20)
Level of education	None	23 (5)
	Primary	198 (46)
	Secondary	169 (39)
	Technical college	34 (8)
	Degree	8 (2)
Occupational status	Formal employment	68 (16)
	Self-employment	131 (30)
	Farmer	174 (40)
	Unemployed	27 (6)
	Housewife	32 (7)
Average household monthly income (USD)	0	8 (2)
	1–27	179 (41)
	27–135	226 (52)
	135–270	13 (3)
	>270	6 (1)
Had a chronic illness	Yes	99 (23)
	No	333 (77)
Owned or had animals in their household	Yes	249 (42)
	No	183 (58)
Animals normally suffered from a seasonal illness	Yes	222 (89)
	No	27 (11)

### Knowledge on substandard and falsified medicines

The majority of respondents (83%) stated that they had ever heard about SFMs although only 31% (110/360) could correctly define them. Only 7% of the respondents accurately identified the correct falsified medicine indicated on the card despite almost a quarter (24%) of respondents stating that they believed they could distinguish SFMs from genuine brands. Respondents mainly recognised SFMs from genuine ones when the pills had a different colour, texture or shape (41%), and if the packaging and label appeared different (34%). When deciding whether or not to purchase a medicine, the respondents reported that they mainly considered the expiry date (29%) and the intended effects of the medicine (27%). When asked whether they knew how to lodge a complaint regarding SFMs, 76% of the respondents reported that they did not know how to do so. Among the respondents, 73% agreed that SFMs could be discovered in the legal medicine supply chain. Most of the respondents (71%) indicated that they were aware of hazards associated with counterfeit medicines including death (56%), liver damage (29%), and rash (26%) (Table 2).

**Table 2.** Knowledge on substandard and falsified medicines.

Variable	Responses	Frequency *n* = 432 (%)
Had heard about SFMs	No	66 (15)
	Yes	360 (83)
	Unsure	6 (1)
Correctly defined SFMs (*n* = 360)	Yes (explained both substandard and falsified medicines correctly)	110 (31)
	Yes (only explained substandard medicines correctly)	63 (18)
	Yes (only explained falsified medicines correctly)	55 (15)
	No	93 (26)
	Unsure	39 (11)
Believed they would be able to recognise SFMs among genuine brands	Yes	105 (24)
	No	327 (76)
Mentioned how SFMs were recognised among other brands^a^ (*n* = 105)	Security seal tampered with	38 (36)
	No hologram / security sticker	25 (24)
	Packaging had a different colour	30 (29)
	Pill had a different colour texture / shape	43 (41)
	Packaging had a different write up / label	36 (34)
	Medicine had diverse side effects	26 (25)
	Medicine did not cure the condition	30 (29)
	Taste of medicine	8 (8)
	Medicine had a low price	23 (22)
	Medicine had no expiry date	30 (29)
Correctly identified which medicine was falsified as per the card provided	No	400 (93)
	Yes	32 (7)
Agreed that branded and generic medicines could be made substandard and falsified or adulterated and sold for profit	No	48 (11)
	Yes	320 (74)
	Unsure	64 (15)
Stated that the quality, efficacy and safety of SFMs was guaranteed	No	191 (44)
	Yes	115 (27)
	Unsure	126 (29)
Agreed with the possibility of medicines for treating chronic illnesses such as heart disease or cancer being counterfeited / adulterated	No	71 (16)
	Yes	278 (64)
	Unsure	83 (19)
Agreed with the possibility of SFMs being discovered in the legal medicine supply chain, for example, through licensed wholesalers and traders	No	67 (16)
	Yes	316 (73)
	Unsure	49 (11)
Knew how to lodge a complaint regarding SFMs	No	330 (76)
	Yes	82 (19)
	Unsure	20 (5)
How they would lodge a complaint concerning SFMs^a^ (*n* = 82)	Report to National Drug Authority	10 (12)
	Report to police	19 (23)
	Report to health workers	56 (68)
	Report to a local leader	23 (28)
The most important factor considered when deciding whether or not to buy a medicine	Name of medicine	45 (10)
	Price of medicine	73 (17)
	Effectiveness of medicine	116 (27)
	Country of origin	6 (1)
	Location of pharmacy	15 (4)
	Did not know	51 (12)
	Expiry date	126 (29)
Awareness of hazards associated with the use of counterfeit medications	No	104 (24)
	Yes	307 (71)
	Unsure	21 (5)
Hazards that could be as a result of counterfeit medications^a^ (*n* = 307)	Cardiovascular problems	29 (10)
	Fever	61 (20)
	Allergy	79 (26)
	Brain damage	51 (17)
	Vomiting	52 (17)
	Liver damage	90 (29)
	Death	171 (56)
	Coma	9 (3)
	Rash	81 (26)
	Worsening of disease	36 (12)
	Others	28 (9)

^a^ Multi-choice response.

### Awareness of substandard and falsified medicines

Less than half of the respondents (42%) had experienced an advertisement or campaign about SFMs in the previous 6 months. Advertisements the respondents reported experiencing on SFMs were mainly through radio 61% (112/183) and television 49% (89/183), and the majority of these adverts targeted human health (61%). Respondents were suspicious that the medicine they purchased was substandard or falsified when it did not cure the condition 53% (76/143) and when it was of a low price 23% (33/143). Over half (52%) of respondents did not use but disposed of medicine that they suspected was substandard or falsified after purchase, while 25% told a health worker at a facility and only 4% reported to the National Drug Authority. When asked about what action should be taken first after purchasing SFMs, 32% of the respondents stated that they would tell a health worker at a facility, and 10% said that suspicions should be reported to the National Drug Authority. Respondents identified multiple entities as being responsible for the presence of SFMs in the market. Almost half (45%) of the respondents mentioned the Ministry of Health and only 5% cited the National Drug Authority (Table 3).

**Table 3.** Awareness of substandard and falsified medicines.

Variable	Responses	Frequency *n* = 432 (%)
Experienced any advertisement or campaign about SFMs in the previous past 6 months	No	243 (56)
	Yes	183 (42)
	Unsure	6 (1)
Place the advertisement or campaign was experienced^a^ (*n* = 183)	Television	89 (49)
	Billboards	3 (2)
	Radio	112 (61)
	Health facilities	14 (8)
	Friends and family	14 (8)
	Social media	17 (9)
	University and schools	1 (1)
Focus of the advertisement or campaign (*n* = 183)	SFMs in human health	112 (61)
	SFMs in animal health	31 (17)
	Both (SFMs in both human and animal health)	40 (22)
Learnt anything from the advertisement (*n* = 183)	Yes	158 (86)
	No	20 (11)
	Unsure	5 (3)
Had ever suspected that the medicine purchased was substandard or falsified^a^	Yes (for themselves)	102 (24)
	Yes (for someone they knew)	54 (13)
	No	287 (67)
Why they suspected it was substandard or falsified^a^ (*n* = 143)	Security seal tampered with	23 (16)
	No hologram / security sticker	10 (7)
	Packaging had a different colour	15 (11)
	Pill had a different colour / texture / shape	32 (22)
	Packaging had a different write-up / label	14 (10)
	Medicine had diverse side effects	37 (26)
	Medicine did not cure the condition	76 (53)
	Taste of medicine	3 (2)
	Medicine had a low price	33 (23)
	Medicine had no expiry date	23 (16)
Actions previously taken after suspecting SFMs^a^ (*n* = 157)	Reported to National Drug Authority	5 (4)
	Checked the package to confirm	10 (7)
	Told another pharmacist at the point of purchase	9 (6)
	Told the health worker at the facility	36 (25)
	Took back medicine to the point of purchase	17 (12)
	Continued using the SFM	12 (9)
	Did not use but kept it	13 (9)
	Did not use and disposed of it	74 (52)
The first action that should be taken if someone suspected that a medicine was substandard or falsified	Return the medicine	92 (21)
	Contact the pharmacist	27 (6)
	Tell the health worker	140 (32)
	Tell a neighbour or friend	9 (2)
	Dispose of the medicine	74 (17)
	Nothing	25 (6)
	Report to National Drug Authority	42 (10)
	Buy a different medicine	23 (5)
Entity / person responsible for the availability of SFMs on the market^a^	Whole sellers	53 (12)
	Pharmacists	52 (12)
	Other health workers	82 (19)
	Manufacturers	60 (14)
	Customs	68 (16)
	Ministry of Health	192 (45)
	National Drug Authority	20 (5)
	Did not know	79 (18)

^a^ Multi-choice response.

### Attitudes towards substandard and falsified medicines

Almost two-thirds (62%) of the respondents disagreed that most human SFMs were as good as genuine human medicines. Similarly, 60% of the participants also disagreed that most animal SFMs were as good as genuine animal medicines. Most respondents agreed (44%) and strongly agreed (39%) in humans, while 49% agreed and 28% strongly agreed in animals that genuine medicines were highly-priced when compared with SFMs. Half (51%) of the respondents strongly agreed and 45% agreed in humans, while 55% agreed and 40% strongly agreed in animals that SFMs could be very dangerous. When asked whether SFMs could be easily identified by their price and quality, 36% agreed and 14% strongly agreed for humans, while 34% agreed and 11% strongly agreed for animals. Most of the respondents agreed (39%) or strongly agreed (55%) that human drug shops and pharmacies that knowingly dispensed SFMs were unethical and unprofessional. Similarly, the majority also agreed (51%) or strongly agreed (42%) that veterinary drug shops and pharmacies that knowingly dispensed SFMs were unethical and unprofessional (Table 4).

**Table 4.** Attitudes towards substandard and falsified medicines for human and animal health.

Variable	Strongly disagree *n* = 432(%)	Disagree *n* = 432(%)	Neutral *n* = 432(%)	Agree *n* = 432(%)	Strongly agree *n* = 432(%)
Most human SFMs were as good as genuine human medicine	117 (27)	267 (62)	27 (6)	17 (4)	4 (1)
Most animal SFMs were as good as genuine animal medicines	120 (28)	259 (60)	37 (9)	16 (4)	0 (0)
Human pharmacies and drug shops had SFMs because their quality was acceptable	111 (26)	200 (46)	74 (17)	36 (8)	11 (3)
Veterinary pharmacies and drug shops had SFMs because their quality was acceptable	100 (23)	188 (44)	107 (25)	31(7)	6 (1)
Many genuine human medicines were highly priced while SFMs were of lower price	10 (2)	28 (7)	34 (8)	191 (44)	169 (39)
Many genuine animal medicines were highly priced while SFMs were of lower price	16 (4)	30 (7)	53 (12)	211 (49)	122 (28)
SFMs could be very dangerous to humans	4 (1)	6 (1)	8 (2)	195 (45)	219 (51)
SFMs could be very dangerous to animals	5 (1)	8 (2)	11 (3)	236 (55)	172 (40)
It was easy to spot human SFMs by their quality and price	50 (12)	59 (14)	106 (25)	156 (36)	61 (14)
It was easy to spot animal SFMs by their quality and price	57 (13)	53 (13)	128 (30)	146 (34)	48 (11)
Had knowingly bought SFMs for humans in the past	156 (36)	241(56)	17 (3.9)	14 (3)	4 (1)
Had knowingly bought SFMs for animals in the past	144 (33)	244 (57)	22 (5)	17 (4)	5 (1)
Human SFMs are easier to differentiate from genuine products than animal SFMs	96 (22)	107 (25)	146 (34)	70 (16)	13 (3)
Animal SFMs are easier to differentiate from genuine products than human SFMs	85 (20)	110 (26)	150 (35)	58 (13)	29 (7)
Human drug shops / pharmacies that knowingly dispense SFMs are unethical and unprofessional	3 (1)	13 (4)	10 (2)	169 (39)	237 (55)
Veterinary drug shops / pharmacies that knowingly dispense SFMs are unethical and unprofessional	3 (1)	9 (2)	15 (4)	222 (51)	183 (42)
The consequences of using SFMs in humans are not as bad as those of using SFMs in animals	118 (27)	158 (37)	110 (25.5)	38 (8.8)	5 (1.9)
The consequences of using SFMs in animals are not as bad as those of using SFMs in humans	111 (26)	134 (37)	121 (28.0)	59 (13.7)	7 (1.6)

### Practices regarding the acquisition and use of substandard and falsified medicines in humans

Respondents reported obtaining medicines for human use when they fell sick mainly from drug shops (52%) and pharmacies in government health facilities (45%). Most respondents (65%) reported that they had never knowingly bought SFMs. Of the respondents (14%) who had previously purchased SFMs, only 5% (3/60) had done so knowingly. Respondents reported various measures used to ensure that the medicine bought was genuine. Seeking health worker advice on the medicine brand (40%) and choosing medicine from a trustworthy pharmacy (34%) were the most common measures. About a quarter of the respondents (26%) checked for information regarding the expiry date when buying medicine for their family or themselves. Almost all of the respondents (98%) had never reported the purchase of SFMs to a concerned authority (Table 5).

**Table 5.** Practices on the acquisition and use of substandard and falsified medicines in humans.

Variable	Responses	Frequency *n* = 432 (%)
Frequency of purchasing / obtaining medicine for human use when one fell sick	Never	4 (1)
	Rarely	36 (8)
	Sometimes	95 (22)
	Most of the times	158 (37)
	Always	139 (32)
Source of human medicine^a^	Drug shop	223 (52)
	Pharmacy in the community	144 (33)
	Pharmacy in a government health facility	193 (45)
	Pharmacy in private clinic / health facility	101 (23)
	Friends and family	5(1)
	Traditional medicine shops	19 (4)
Ever bought any product suspected to be SFM before	No	280 (65)
	Yes	60 (14)
	Unsure	92 (21)
The SFM was bought knowingly (*n* = 60)	No	57 (95)
	Yes	3 (5)
Measures taken to ensure that the medicine being bought for oneself or family was genuine^a^	Seeking health worker advice for choosing the medicine brand	147 (40)
	Getting the medicine from a trustworthy pharmacist / drug shop attendant	126 (34)
	Buying medicine manufactured outside Uganda	8 (2)
	Buying medicine that has worked in the past	37 (10)
	Buying from an authorised drug shop	118 (32)
	Expiry date	95 (26)
	None	50 (13)
Ever reported SFMs to the concerned authority	No	422 (98)
	Yes	7 (2)
	Unsure	3 (1)
Name of authority / person reported to (*n* = 7)	Health worker	7 (100)
Checked for information regarding the expiry date when buying medicine for family or themselves	Never	57 (13)
	Rarely	67 (16)
	Sometimes	95 (22)
	Most times	105 (24)
	Always	108 (25)

^a^ Multi-choice response.

### Practices regarding the acquisition and use of substandard and falsified medicines in animals

Of the 42% of the respondents who owned animals, 25% always and 35% most times purchased medicine for the treatment of sick animals, mainly from a veterinary pharmacy in the community (57%). Almost a quarter (24%) of the respondents reported having ever bought SFMs which they mainly differentiated from genuine ones when the medicines did not cure the condition (66%). Almost all the respondents (97%) had never reported the purchase of SFMs for animals to a concerned authority. Seeking a veterinary officer’s advice for choosing the medicine brand (43%) and getting medicine from a trustworthy veterinary pharmacist (29%) were the most practiced measures to ensure that medicine bought for animals was genuine. Many respondents mentioned that they never (35%) or rarely (12%) checked for information regarding the expiry date when they bought medicine for animals including poultry (Table 6).

**Table 6.** Practices on the acquisition and use of substandard and falsified medicines in animals.

Variable	Responses	Frequency *n* = 249 (%)
Frequency of purchasing / obtaining medicine for use when animals fell sick	Never	30 (12)
	Rarely	23 (9)
	Sometimes	47 (19)
	Most times	87 (35)
	Always	62 (25)
Source of medicine for animals^a^	Drug shop	57 (23)
	Veterinary pharmacy in the community	141 (57)
	Veterinary pharmacy in government health facility	27 (11)
	Pharmacy in a private clinic / hospital	40 (16)
	Fellow farmers and friends	10 (4)
	Traditional medicine shops	17 (7)
	Veterinary worker	20 (8)
Ever bought any SFMs for their animals	No	153 (61)
	Yes	59 (24)
	Unsure	37 (15)
Ways of differentiating SFMs for animal from genuine ones^a^ (*n* = 59)	The security seal had been tampered with	7 (12)
	It did not have a hologram	3 (5)
	It had a different colour of the packaging	1 (2)
	It had a different pill (colour / shape / texture)	5 (9)
	It had a different write up / label (unusual font sizes, spelling errors)	3 (5)
	Was told by the drug shop / pharmacy attendant	4 (7)
	Adverse / side effects of the medicine	15 (25)
	Did not cure the condition	39 (66)
	Low price of the medicine	10 (17)
	Expiry date	6 (10)
Measures taken to ensure that the medicine bought for animals was genuine^a^	Seeking veterinary officer’s advice for choosing the medicine brand	107 (43)
	Getting the medicine from a trustworthy veterinary pharmacist	72 (29)
	Buying medicine manufactured outside of Uganda	4 (2)
	Buying medicine that has worked in the past	11 (4)
	Buying from an authorised veterinary drug shop / pharmacy	28 (11)
	Expiry date	47 (19)
	None	58 (23)
	Getting advice from a fellow farmer	22 (9)
Ever reported SFMs in animals to the concerned authority	No	242 (97)
	Yes	6 (2)
	Unsure	1(0)
Checked for information regarding the expiry date when they bought medicine for animals including poultry	Never	87 (35)
	Rarely	29 (12)
	Sometimes	53 (21)
	Most times	46 (19)
	Always	34 (14)

^a^ Multi-choice response.

## Discussion

The study found that community members from a range of backgrounds generally had limited knowledge and related poor practices despite commendable attitudes on SFMs. Most respondents lacked the ability to correctly identify SFMs or check purchased medications for their authenticity. This can result in unintentional and intentional harmful practices when buying and using medicines for both humans and animals. Many study respondents reported never having purchased and used SFMs knowingly or unknowingly, although a good number suspected that a medicine they previously purchased had been substandard or falsified. In addition, most respondents were unaware of reporting procedures and regulatory bodies in charge of SFMs in Uganda. The study findings highlight the gap in knowledge and practices on SFMs which is a major concern in Uganda, as is the case in different regions of the world (El-Dahiyat et al., 2021). To minimise potential health effects of SFMs on humans and animals including the growing burden of AMR locally and globally, there is need to increase awareness on this public health challenge among various stakeholders including the community (Aminu et al., 2017).

Most respondents in our study had minimal knowledge of SFMs, although they had an idea of what they might be. Despite the majority of respondents (83%) reporting a working knowledge of SFMs, it was not enough to correctly define them. Although a quarter (24%) of our study respondents believed that they could identify SFMs, only 7% accurately identified the SFM when shown pictures of genuine and falsified drug packaging. This implies that more than 90% of the respondents were at risk of inadvertently purchasing and using SFMs. Despite alerts by the National Drug Authority (NDA) about such drugs, information may not reach communities to increase their awareness of SFMs in Uganda. These findings are consistent with those of related studies which show little public awareness about SFMs. For example, a study in Lebanon showed over 93% of participants reported having some knowledge of SFMs, although 29% did not feel confident to be able to correctly distinguish them from genuine ones (Sholy & Saliba, 2018). Similarly, an online study on public awareness of SFMs in different regions of the world revealed that only 31% of the participants could accurately identify SFMs (El-Dahiyat et al., 2021). These findings emphasise the importance of increasing public awareness creation campaigns to enhance knowledge and reduce vulnerability to SFMs.

The inability of respondents in our study to identify SFMs could be due to their close resemblance to genuine medicines (Gurney et al., 2017; WHO, 2018). Indeed, most respondents may not have been able to correctly identify SFMs if they had the same colour or label as genuine ones. This is because nearly half of the respondents in our study (41%) expected SFMs to have colours that were different from those of genuine ones, while 34% expected them to have different labelling. Similar findings were obtained in a study conducted in Sudan where 30% of participants used differences in packaging as an indicator of a medical product being substandard or falsified (Wagiella et al., 2020). This implies that any SFM that had the same colour, packaging or labelling as a genuine medicine would not easily be recognised. With manufacturers and vendors of counterfeit medications frequently spending a lot of money to refine their appearance and labels to look exactly like genuine ones (WHO, 2017), it may be difficult to identify SFMs using only colour differentiation and labelling as determinants alone. Over a quarter of respondents in our study (29%) reported dependence on expiry dates as an indicator of SFMs. This misconception could lead community members to unknowingly purchase SFMs if the lengthy expiry date misleads the consumer into thinking the medicine is legitimate.

Most respondents in our study (73%) were of the view that SFMs could be found in the legal medicine supply chain. This finding is in line with related studies which reported the global proliferation of SFMs in legal supply chains in LMICs (Mackey et al., 2015) including Ethiopia (Worku et al., 2024), Egypt (Wagiella et al., 2020), and China (Newton et al., 2014). These findings suggest non-adherence to international regulations, weak law enforcement systems (Orubu et al., 2020), and unsupervised (medical) supply chains (Islam & Islam, 2022). In addition, high pressures on healthcare facility managers to buy medication solely based on price, rather than a focus on quality (Glass, 2014) encourages the purchase of SFMs. High poverty levels and low-purchasing power of consumers in LMICs are drivers for the purchase of cheaper medications which may be substandard or falsified. Other possible reasons for the presence of SFMs in the formal supply chains could be unavailability and unaffordability of vital medicines in sub-Saharan African countries (De Terline et al., 2018; Renschler et al., 2015) and other LMICs (Ware et al., 2023). Hence, the availability of SFMs in legal supply chains in these settings could be to meet the demand. It is important to note that increased presence of SFMs in high income countries has been seen via online pharmaceutical vendors since this is more convenient for consumers than obtaining medicines legally through a prescription (O'Hagan & Garlington, 2018; Ofori-Parku, 2022; Orizio et al., 2011; WHO, 2017). This concern could emerge in LMICs in the future with the increasing trend of online business in these settings.

The majority of respondents in our study (71%) were aware of the dangers of using SFMs. This is slightly higher than in a related study in several countries in Europe, Asia, Africa, America and Middle East which showed 52% of respondents reporting awareness of the dangers of SFMs (El-Dahiyat et al., 2021). Our study findings further revealed more than half (56%) of the respondents were aware that SFMs could cause premature death, consistent with findings among pharmacy professionals in Ethiopia (Worku et al., 2024). However, many respondents also lacked awareness about the specific dangers to health of SFMs. There is a high likelihood that people with low awareness of the risks associated with SFMs could purchase and subsequently use them. Therefore, there is need for consumers and regulatory agencies to work together to identify, expose, and prosecute perpetrators of SFMs. This could be achieved by implementation of the WHO mechanisms for reporting SFMs within formal and informal supply chains globally (WHO, 2017). Locally, NDA, the body mandated to ensure the availability of efficacious medicines to the population in Uganda, should increase efforts to reduce SFMs on the market to protect public health.

Our findings suggest minimal publicity on SFMs as only 42% of the respondents reported having experienced a related advert within the previous 6 months. This is contrary to findings from a study conducted in Tanzania which showed 69% of participants citing the media as a resource for knowledge about fake medications (Mhando et al., 2016). These differences could be contextual as most of our study respondents came from rural (51%) and semi-urban settlements (32%), whereas those in the Tanzanian study all came from the city of Mwanza who likely had better access to information on SFMs including from the media. Other studies have reported SFM awareness via social media (Al-Worafi, 2020; Wagiella et al., 2020) which could be explored further in Uganda and other LMICs. Among the 183 respondents who had experienced advertisements on SFMs in our study, the majority (61%) reported hearing about them on radio, while 49% saw them on television. Therefore, individuals who do not have access to either radio or television sets may not get the available information about SFMs in the study setting. In addition, most (61%) advertisements seen were reported to be concentrated on humans rather than consideration for this problem in the animal health sector too. Thus, there is a likelihood that people may unknowingly use SFMs among their animals leading to poor health effects, including AMR, and their possible transference to humans including consumers of animal products. This gap in public literacy on SFMs among animals is of concern particularly in this era where the One Health approach is considered essential to combatting the growing threat of AMR (White & Hughes, 2019). Emphasis on the One Health approach would necessitate multidisciplinary and multisectoral stakeholder collaboration between human, animal and environment professionals.

In our study, 32% of the respondents mentioned that they would report to a health worker at a facility if they suspected a medication to be substandard or falsified. Although this could be a good first step, it may not be feasible for people who have little access to qualified health practitioners as evidence suggests inadequate health workforce globally, especially in rural areas in sub-Saharan African countries (Ahmat et al., 2022) including Uganda. In addition, reporting suspected SFMs to a health worker may not yield the intended results if they are not empowered with adequate knowledge and skills to respond adequately. Ideally, the appropriate regulatory bodies should be engaged in addressing the dangers of SFMs including the NDA in Uganda whose primary role includes overseeing the supply chain of medicines through the National Drug Policy and Authority Act (NDPA, 2000). However, most respondents were unaware of the correct regulatory bodies to report any instances of SFMs to, as nearly half (45%) mentioned the Ministry of Health, and only 5% correctly cited NDA. Thus, more community-based campaigns, including through the media, are required to create awareness on the regulatory authorities in charge of SFMs, their mandate, and how to report suspected SFMs to them.

Nearly all respondents in our study agreed / strongly agreed (96%) in humans and 94% agreed / strongly agreed in animals that SFMs pose a risk to health. Despite this knowledge about the dangers of SFMs to human and animal health, about half of respondents were confident to be able to detect SFMs using both indicators of price and quality. When asked whether SFMs could be easily identified by their price and quality, 51% agreed / strongly agreed for human SFMs, while 45% agreed / strongly agreed for animal SFMs. In addition, the majority of respondents agreed / strongly agreed (83%) in humans and 77% agreed / strongly agreed in animals that genuine medical products have higher prices than SFMs. Although earlier research showed that some SFMs may have slightly lower prices than genuine medical products (Kovacs et al., 2014), it may not always be the case as some can have the same price as genuine products. Thus, there is a possibility that although SFMs may have incorrect or no active ingredients (WHO, 2018), many individuals could mistake them for genuine medicines if they have similar prices as genuine products. A possible remedy is for more public education on detecting SFMs using other measures beyond price such as visual inspection (Aminu et al., 2017).

One of the limitations of this study is that it relied on self-reported data hence the likelihood of recall and social desirability bias among the respondents. The study was descriptive hence factors associated with practices and actions related to SFMs were not assessed. Including a design effect in the sample size calculation could have also increased the sample size. In addition, the study was conducted in only one district hence the findings may not be generalisable to other parts of the country. Nevertheless, this is one of the few studies that have been carried out on SFMs in Uganda for both humans and animals. Therefore, the evidence generated could be instrumental in informing best practices regarding SFMs not only in humans but also for animals.

## Conclusion

There was generally limited knowledge and associated poor practices regarding SFMs for both humans and animals despite commendable attitudes. In particular, the majority of participants were unable to identify SFMs and unaware of the correct reporting procedures if they suspected SFMs. There is a need for key stakeholder engagement to increase awareness on SFMs to minimise the potential risks to health among the community. Key stakeholders such as health and regulatory authorities should intensify efforts to understand the barriers to safe medicines use and reduce the availability of SFMs in humans and animals hence improving access to good quality medicine products to protect public health.

## Supplementary Material

Supplemental Material

## Data Availability

The data sets used and/or analysed during the current study are available from the corresponding author upon reasonable request.
