# Supplementary figures and images for: Knowledge, attitudes and practices on substandard and falsified medicines for human and animal use in Wakiso district, Uganda
Source: J Pharm Policy Pract. 2025 Oct 6;18(1):2564822. doi: 10.1080/20523211.2025.2564822 (PMC12502104; doi:10.1080/20523211.2025.2564822)

**Supplementary File 1: Illustration of the card used for identification of SFMs**


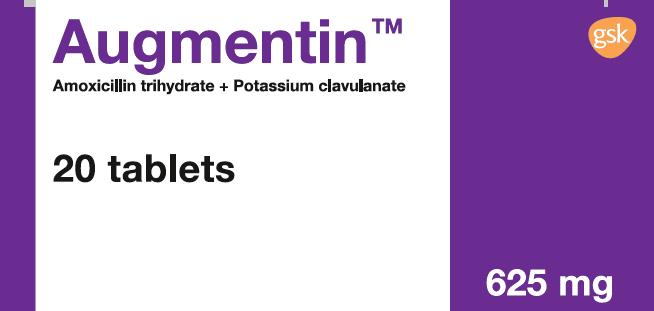


Image A


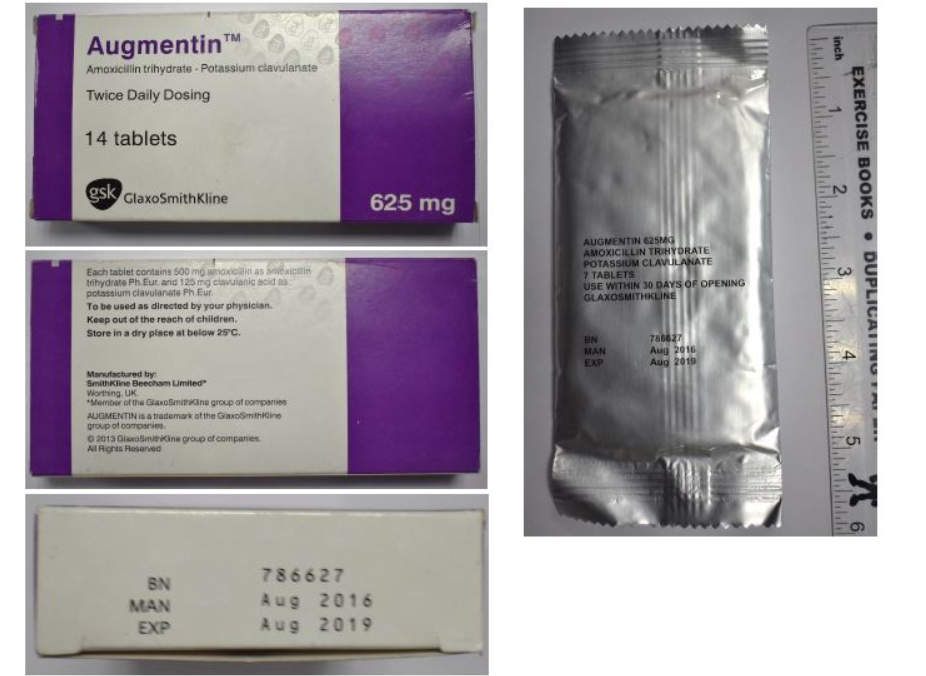


Image B

Supplement: Supplemental Material [file JPPP_A_2564822_SM6786.docx]
